# *EIF2B2* mutations in vanishing white matter disease hypersuppress translation and delay recovery during the integrated stress response

**DOI:** 10.1261/rna.066563.118

**Published:** 2018-06

**Authors:** Stephanie L. Moon, Roy Parker

**Affiliations:** 1 Department of Chemistry and Biochemistry, University of Colorado, Boulder, Colorado 80303, USA; 2 Howard Hughes Medical Institute, University of Colorado, Boulder, Colorado 80303, USA

**Keywords:** white matter disease, eIF2B, GADD34, integrated stress response, ISRIB

## Abstract

Mutations in eIF2B genes cause vanishing white matter disease (VWMD), a fatal leukodystrophy that can manifest following physical trauma or illness, conditions that activate the integrated stress response (ISR). EIF2B is the guanine exchange factor for eIF2, facilitating ternary complex formation and translation initiation. During the ISR, eIF2α is phosphorylated and inhibits eIF2B, causing global translation suppression and stress-induced gene translation, allowing stress adaptation and recovery. We demonstrate that VWMD patient cells hypersuppress translation during the ISR caused by acute ER stress, delaying stress-induced gene expression and interrupting a negative feedback loop that allows translational recovery by GADD34-mediated dephosphorylation of phospho-eIF2α. Thus, cells from VWMD patients undergo a prolonged state of translational hyperrepression and fail to recover from stress. We demonstrate that small molecules targeting eIF2B or the eIF2α kinase PERK rescue translation defects in patient cells. Therefore, defects in the ISR could contribute to white matter loss in VWMD.

## INTRODUCTION

Vanishing white matter disease (VWMD), a fatal leukodystrophy in children and adults, is caused by mutations in any of the five *EIF2B* genes (Leegwater et al. 2001). VWMD patients often experience episodes of progressive white matter loss following trauma or febrile illnesses (Leegwater et al. 2001). Physical trauma and neuroinflammation can cause ER stress in the brain and activate the integrated stress response (ISR), a pathway that causes global translational suppression and the expression of stress-induced genes (Petrov et al. 2001; Lin et al. 2005; Begum et al. 2014; Chou et al. 2017). EIF2B is an important translation initiation factor that is targeted early in the ISR to mediate the translational repression arm of this pathway. This suggested the hypothesis that disease-causing mutations in *EIF2B* genes alter the cellular response to stress by perturbing the regulation of translation during the ISR.

The eIF2B complex is a dimer of heteropentamers (Gordiyenko et al. 2014; Kashiwagi et al. 2016) that exchanges GDP for GTP on eIF2 to allow ternary complex formation and translation initiation. During stresses such as viral infections and ER stress, the eIF2 complex is targeted by stress-activated protein kinases that phosphorylate eIF2α (Hinnebusch 1994). Phosphorylated eIF2α binds the eIF2B complex with higher affinity and represses its exchange activity, leading to reduced ternary complex formation and global translation suppression (Wek et al. 2006). Because fewer translation initiation events take place during the ISR, stress-induced genes that are regulated through upstream open reading frames (uORFs) are selectively translated. These stress-induced genes include *ATF4* and *CHOP*, transcription factors that promote the expression of genes including the eIF2α phosphatase *GADD3*4, which is one of the feedback mechanisms that facilitate the recovery from stress in the second phase of the ISR (Kojima et al. 2003; Han et al. 2013; Pakos-Zebrucka et al. 2016). As GADD34 is also translationally regulated through uORF mechanisms (Lee et al. 2009), the ISR is governed by coordinated changes in both transcriptional and translational control mechanisms. The coordinated arms of the ISR that facilitate translational repression and stress-induced gene expression are therefore both important for cell survival of stress, and defects in ISR pathway components can cause neurodevelopmental and/or neurodegenerative disorders (Borck et al. 2012; Bruch et al. 2015; Kernohan et al. 2015).

Previous work suggests that defective maturation and cytopathology of astrocytes and oligodendrocytes, the myelinating cells of the brain, may be the cellular basis of VWMD (van der Voorn et al. 2005; van Kollenburg et al. 2006a; Bugiani et al. 2011). Markers of stress-induced translational suppression are elevated in glial cells in VWMD (van der Voorn et al. 2005; van Kollenburg et al. 2006a), and constitutive induction of stress-response pathways in oligodendrocytes recapitulates many aspects of VWMD in a mouse model (Lin et al. 2014). These observations suggest that aberrant stress responses may underlie glial cytopathology in VWMD. However, how disease-causing mutations in VWMD impact the acute stress response is not clear.

We undertook this study to determine if VWMD-causing mutations in *EIF2B2*, a regulatory subunit of the eIF2B complex, interfered with the cellular response to acute stress, perhaps explaining a key aspect of VWMD: the progressive, episodic loss of white matter in response to trauma or illness. We tested this hypothesis by assessing the translation activity and induction of stress response genes in cell lines derived from VWMD patients and matched controls. Our findings suggest that mutations in *EIF2B2* causative of VWMD uncouple the two arms of the ISR by hypersuppressing translation initiation activity during stress, suppressing stress-induced genes including *GADD34* to disrupt a negative feedback loop necessary for the adaptation to and recovery from stress.

## RESULTS

### VWMD mutations in *EIF2B2* do not decrease bulk translation under normal conditions

Because the eIF2B complex is important for both normal translation activity and regulation of translation activity during stress, *EIF2B* mutations causative of VWMD could cause a global reduction in translation activity, and/or perturb the integrated stress response. We addressed these possibilities as follows using three immortalized lymphoblast cell lines derived from patients with VWMD caused by mutations in the *EIF2B2* subunit, as compared to three age-, sex- and ethnicity-matched control cell lines (described in Materials and Methods).

**FIGURE 1.**
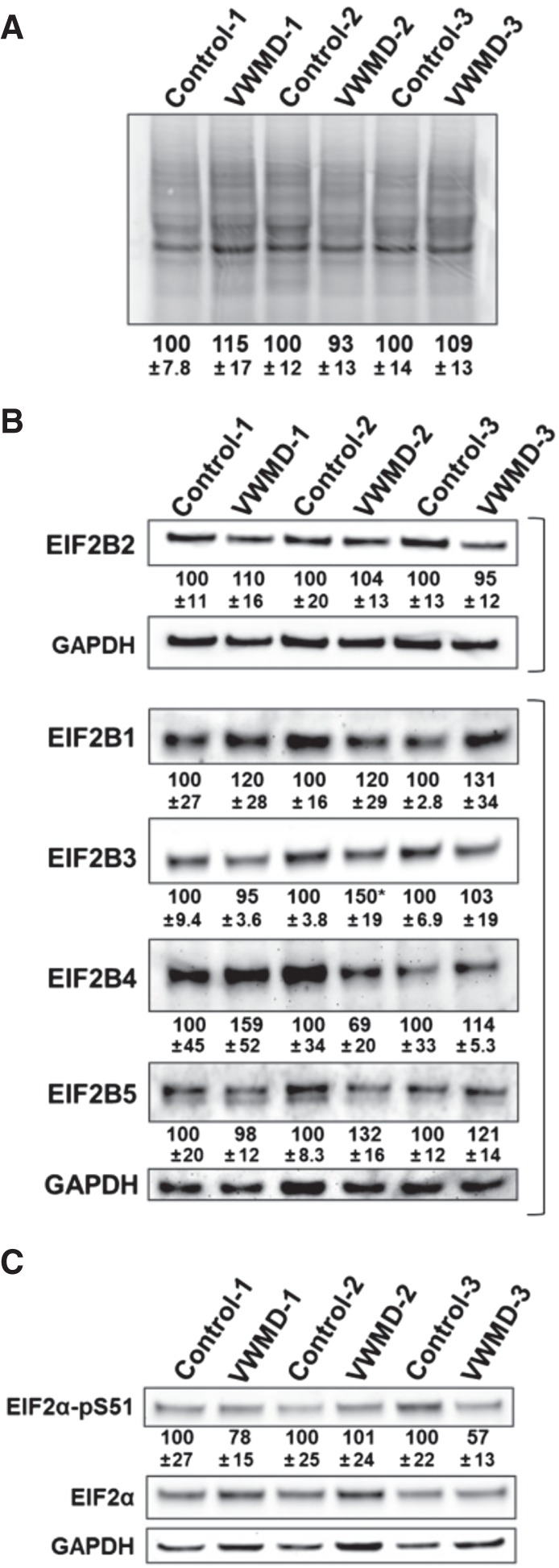
VWMD cell lines with *EIF2B2* mutations have normal translation activity in unstressed conditions. (*A*) Equal numbers of control and VWMD patient cell lines were fed ^35^S-labeled met and cys, lysed and proteins separated on 4%–12% NuPAGE SDS PAGE gels and detected by phosphorimaging. The intensity of each lane was quantified and the averages ±SD of the lane intensity of each VWMD patient sample relative to the matched control from three independent experiments is shown *below* a representative gel. (*B*) The levels of EIF2B1, EIF2B2, EIF2B3, EIF2B4, and EIF2B5 proteins in VWMD patient and control lymphoblasts was assessed by western blotting. Brackets enclose blots for which the indicated EIF2B protein was stripped and reprobed for GAPDH as a loading control. Quantification shows the average relative EIF2B protein levels from three independent experiments ±SEM *below* a representative blot. (*C*) Phospho-eIF2α levels were determined by western blotting in VWMD patient and matched controls. The average abundance of phospho-eIF2α (“EIF2α-pS51”) relative to total EIF2α from three independent experiments ±SEM is shown *below* a representative blot. Student's *t*-test was used to assess significance in VWMD samples versus control for all experiments.

Several observations demonstrated that VWMD cell lines had similar levels of bulk translation as control cells in the absence of stress. First, by measuring the incorporation of labeled amino acids into nascent polypeptides, we determined that translation in the absence of stress in control and VWMD cell lines was similar (Fig. 1A). This is similar to previous observations that VWMD cell lines show normal levels of translation in the absence of stress (Kantor et al. 2005; van Kollenburg et al. 2006b; Sekine et al. 2016; Wong et al. 2018). Second, western blot analysis showed the examined *EIF2B2* mutations did not reduce the levels of EIF2B2 protein (Fig. 1B) or lead to any consistent changes in the levels of EIF2B1, EIF2B3, EIF2B4, and EIF2B5 proteins (Fig. 1B). Third, using antibodies against phospho-eIF2α we demonstrated that control and VWMD cell lines had very low and similar levels of phospho-eIF2α in the absence of stress (Fig. 1C). These observations argue that VWMD mutations do not perturb translation or cause a constitutive stress response in the absence of stress.

### VWMD patient cell lines hypersuppress translation during acute stress

We then determined how VWMD mutations affected translation activity during an acute stress response when eIF2B-GTP exchange activity is also suppressed by the phosphorylation of eIF2α, and this step becomes limiting for translation initiation activity in the cell. We first measured the degree of translation repression of the control and VWMD cell lines utilizing thapsigargin as a stressor. Thapsigargin induces calcium release from the ER (Thastrup et al. 1990; Lytton et al. 1991), triggering the unfolded protein response (UPR) (McCormick et al. 1997; Pahl 1999), and phosphorylation of eIF2α by PERK. This is a relevant stress since in VWMD patients, glial cells in the white matter exhibit up-regulated markers of the UPR including phosphorylated PERK and phospho-eIF2α (van der Voorn et al. 2005; van Kollenburg et al. 2006a). Further, traumatic brain injury and interferon-γ resulting from neuroinflammation, conditions that trigger white matter loss in VWMD (Leegwater et al. 2001), can cause phosphorylation of eIF2α and activation of the ISR (Petrov et al. 2001; Lin et al. 2005; Begum et al. 2014; Chou et al. 2017).

**FIGURE 2.**
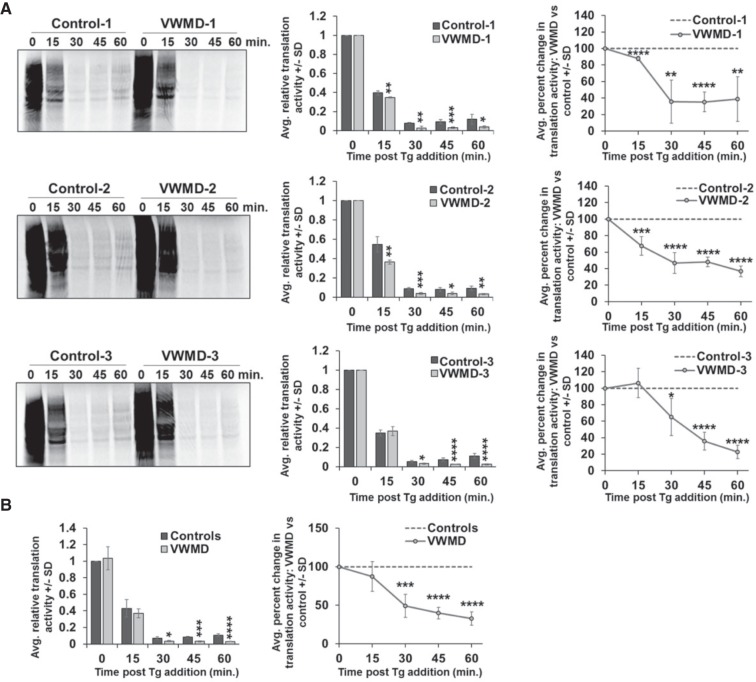
Cells from VWMD patients hypersuppress translation during acute stress. (*A*) Equal numbers of cells from VWMD patient cells and matched controls were treated with 1 µM thapsigargin for 0, 15, 30, 45, or 60 min and were pulse labeled for 15 min with ^35^S-met and -cys prior to sample collection. Representative images are shown at *left* with average translation activity relative to the unstressed time point ±SD of three experiments presented in the bar graphs. The line graphs represent the average ±SD of the percent difference in translation activity at each time point in the indicated VWMD patient cell line relative to the matched control cell line. (*B*) The average ±SD of the translation activity from all control and all VWMD patient cell lines relative to the control unstressed condition is depicted in the bar graph at *left* with the pooled average percent difference in translation activity ±SD shown in the line graph at *right*. Student's *t*-test was done to test significance between control and VWMD samples at each time point, with (*) indicating *P* ≤ 0.05, (**) *P* ≤ 0.01, (***) *P* ≤ 0.005, and (****) *P* ≤ 0.001.

**FIGURE 3.**
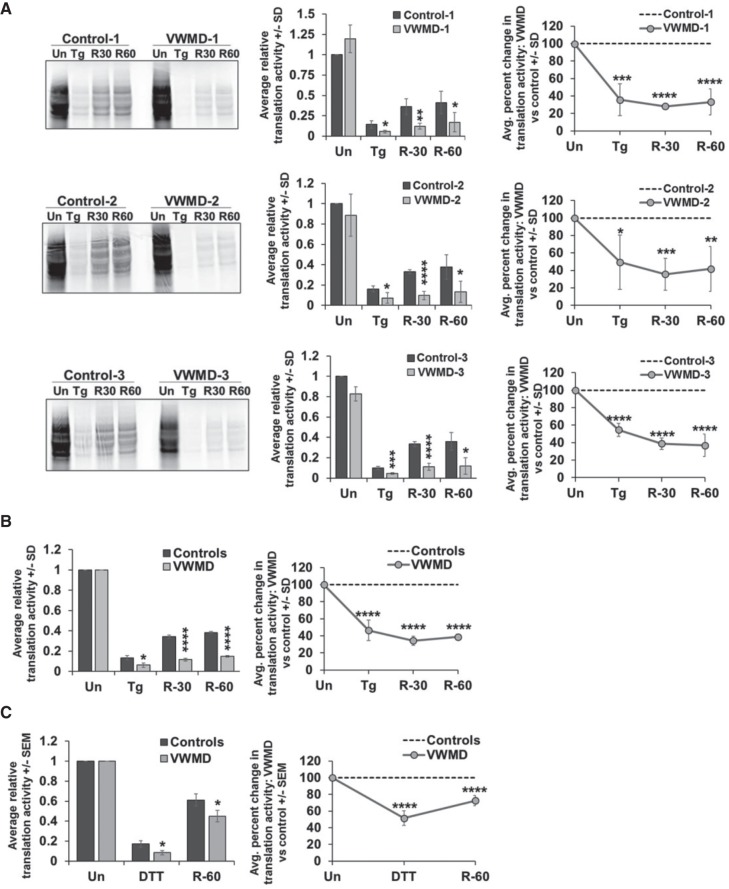
VWMD patient cells have limited recovery of translation activity after acute ER stress. (*A*) Equal numbers of cells from matched controls and VWMD patients were untreated (“Un”), treated with 1 µM thapsigargin for 1 h (“Tg”), or treated with thapsigargin for 1 h, then washed twice and allowed to recover for 30 (“R-30”) or 60 (“R-60”) min. Representative phosphorimages are shown at *left*, with bar graphs depicting average ±SD relative to the control unstressed condition and line graphs showing the average ±SD in percent change in translation activity at each time point in VWMD patient cells relative to control cells. (*B*) The average ±SD of the translation activity in all pooled VWMD and control cells relative to the unstressed “Un” condition is shown in the bar graph, and the line graph represents the average percent difference ±SD of the pooled translation activity in the VWMD patient samples relative to controls in each condition. (*C*) Equal numbers of cells from VWMD patients and matched controls were untreated (“Un”) or treated with 2 mM DTT for 1 h and collected (“DTT”) or washed twice and allowed to recover for 60 min (“R-60”). Cells were fed ^35^S met and cys for 30 min prior to collection. The average translation activity and percent difference in translation activity ±SEM is shown from the pooled VWMD and patient cell lines as in *B*, with the results from the individual matched VWMD and control pairs shown in Supplemental Figure S2. Results represent three to four independent experiments. Student's *t*-test was used to assess significance between VWMD and control samples, with (*) indicating *P* ≤ 0.05, (**) *P* ≤ 0.01, (***) *P* ≤ 0.005, and (****) *P* ≤ 0.001.

An important observation is that all three VWMD patient cell lines repressed translation to a greater degree than control cell lines after 30, 45, and 60 min in the presence of 1 µM thapsigargin (Fig. 2A), and the averages of all controls and VWMD cell lines showed greater repression in the VWMD cell lines (Fig. 2B). Similar results are also seen with lower levels of thapsigargin (Supplemental Fig. S1A,B). We also observed that the VWMD cell lines showed stronger translation repression when the UPR is triggered by DTT treatment (Fig. 3C; Supplemental Fig. S2), or arsenite treatment, which causes eIF2α phosphorylation through the kinase HRI and does not trigger the UPR (Supplemental Fig. S1C,D). These observations indicate that cell lines with *EIF2B2* mutations derived from VWMD patients hyperrepress translation during the ISR when EIF2B activity is inhibited by phospho-eIF2α.

Interestingly, the difference in translation activity between VWMD and control cell lines was greater at longer time points post-stress (Fig. 2A,B), suggesting that the VWMD cells could be deficient at triggering the negative feedback loops in the ISR that allow for translation recovery.

### VWMD patient cell lines display reduced translation activity during the recovery from acute stress

One key aspect of the ISR is that it is reversible. The translation that persists when eIF2α is phosphorylated allows for stress specific translation of ATF4 and other mRNAs, leading to the production of both proteins and the transcription of mRNAs required for recovery from stress. One important stress-induced mRNA is from the *GADD34* gene, which produces a phosphatase that acts in a negative feedback loop to dephosphorylate eIF2α to contribute to translation resumption (Novoa et al. 2001, 2003; Kojima et al. 2003). Therefore, we hypothesized that the hyperrepression of translation in the VWMD cell lines would limit the induction of GADD34 and other proteins required for stress recovery. To examine this possibility, we assessed the ability of VWMD patient cells to resume translation activity following recovery after washing out thapsigargin or DTT after 1 h of acute ER stress to determine if *EIF2B2* mutations limited recovery from stress.

We observed that VWMD patient cells had a significant defect in translation recovery when measured 30 or 60 min after washing out thapsigargin compared to matched controls (Fig. 3A,B). On average, wild-type control cells recovered 38 ± 2.7% translation activity and VWMD cells recovered only 14.9 ± 0.9% translation activity (an ∼2.6-fold reduction) 60 min after thapsigargin stress. Furthermore, VWMD cell lines also exhibited delayed recovery from stress induced by DTT (Fig. 3C; Supplemental Fig. S2). Taken together, these results indicate that the hypersuppression of translation observed in VWMD patient cells during acute ER stress diminishes the recovery of translation after stress, making the ISR less reversible.

### GADD34 expression is delayed in VWMD patient cell lines and associated with prolonged eIF2α phosphorylation

**FIGURE 4.**
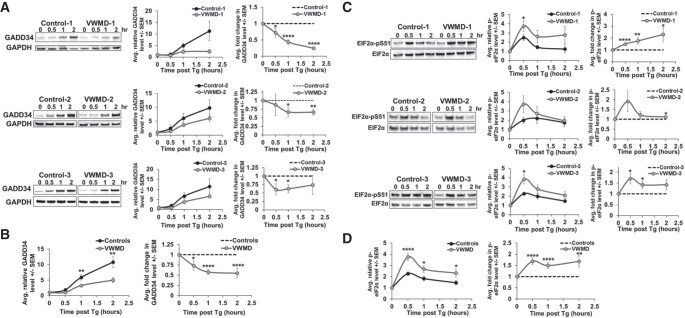
The induction of the stress-induced phosphatase GADD34 is delayed, and levels of its target phospho-eIF2α are increased in VWMD patient cell lines during thapsigargin stress. Equal numbers of VWMD and control cell lines were treated with 1 µM thapsigargin for 0, 0.5, 1, or 2 h. Western blotting was done to detect GADD34 and the loading control GAPDH (*A*,*B*) or phospho-eIF2α and total eIF2α (*C*,*D*). Representative blots are shown, and the average abundance or fold change in GADD34 (*A*) or phospho-eIF2α (*C*) ±SEM from three to four independent experiments are depicted in the graphs at *right*. The pooled average levels and fold change in GADD34 (*B*) or phospho-eIF2α (*D*) in all controls and VWMD patient cell lines are shown ±SEM. Results represent three to four independent experiments, and Student's *t*-test was used to assess significance between VWMD and control samples, with (*) indicating *P* ≤ 0.05, (**)*P* ≤ 0.01, (***)*P* ≤ 0.005, and (****)*P* ≤ 0.001.

The defect in recovery from stress seen in the VWMD cells could be explained by a failure to induce the downstream aspects of the ISR, including the GADD34 protein that would dephosphorylate eIF2α and be one of the feedback loops that contributes to restoring translation. This interpretation predicts that GADD34 protein production should be reduced or delayed in the VWMD cell lines, as it is up-regulated both at the transcription and translation levels during stress (Novoa et al. 2001; Ma and Hendershot 2003; Lee et al. 2009). In addition, due to reduced levels of GADD34, phosphorylated eIF2α should be expected to be higher and/or persist longer in the VWMD cell lines. To test these predictions, we examined the levels of the stress-induced GADD34 protein by western blotting during thapsigargin stress. We observed a delay in the induction and/or reduction in the amount of GADD34 protein induced by thapsigargin stress in all three VWMD cell lines (Fig. 4A). Comparing the averages of all three cell lines and controls also showed a significant reduction in GADD34 levels in the VWMD cell lines (Fig. 4B). The levels of GADD34 were not significantly different in unstressed conditions in VWMD patient cell lines relative to control cell lines.

The reduced GADD34 induction in the VWMD cell lines corresponded with a larger increase, and prolonged persistence, in its target phospho-eIF2α during thapsigargin stress (Fig. 4C,D). Therefore, increased phospho-eIF2α levels could contribute to failure of these cells to maintain some translation activity during and after acute stress. Of note, loss-of-function mutations in *GADD34* also increase phospho-eIF2α levels and block stress-induced gene expression (Novoa et al. 2003). Thus, *EIF2B2* mutations causative of VWMD uncouple the translation and stress response gene induction arms of the ISR by hyperrepressing translation and limiting or delaying stress-induced gene expression.

### Pharmacological rescue of translation defects in cells derived from VWMD patients

**FIGURE 5.**
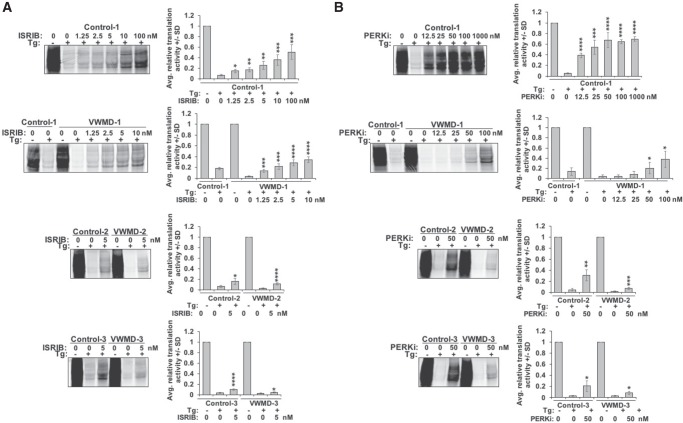
Chemical modulators of eIF2B and PERK activity rescue the hypersuppression of translation in VWMD patient cells during stress. (*A*) Control-1 and VWMD-1 lymphoblasts were incubated with (“+”) or without (“−”) 1 µM thapsigargin (“Tg”) in the presence or absence of ISRIB at 0, 1.25, 2.5, 5, 10, or 100 nM for 1 h and were labeled with ^35^S-met and -cys for 30 min prior to lysis and separation on SDS-PAGE gels and phosphorimaging (*top* two panels). Cells from Control-2, VWMD-2, Control-3, and VWMD-3 were treated as above, only using 0 or 5 nM ISRIB (*bottom* two panels). (*B*) The PERK inhibitor GSK2606414 (“PERKi”) was tested at 0, 12.5, 25, 50, 100, or 1000 nM as in *A* in Control-1 and VWMD-1 lymphoblasts (*top* two panels). Cells from Control-2, VWMD-2, Control-3, and VWMD-3 were treated as above using 0 or 50 nM PERKi (*bottom* panels). Representative phosphorimages are shown at *left* and the graphs depict the average ±SD from three experiments. Student's *t*-tests were done by comparing the drug-treated samples with the samples treated with thapsigargin alone, with (*) indicating *P* ≤ 0. 05; (**) *P* ≤ 0.01, (***) *P* ≤ 0.005, and (****) *P* ≤ 0.001.

The hyperrepression of translation due to the *EIF2B2* mutations in the VWMD cell lines might contribute to disease phenotypes (see Discussion). Given this, we tested if compounds targeting aspects of the ISR could restore translation in the VWMD cell lines to levels seen in normal cells during an acute stress response. For this experiment, we examined ISRIB, which targets eIF2B to increase its guanine exchange activity on eIF2 (Sidrauski et al. 2013; 2015; Sekine et al. 2015) and the PERK inhibitor I (PERKi) (GSK2606414) (Axten et al. 2012). Both ISRIB and PERKi significantly increased translation activity during thapsigargin-mediated ER stress in a dose-dependent manner, although neither drug allowed full recovery of translation activity at the highest concentrations tested (ISRIB at 100 nM and PERKi at 1 µM) in both VWMD and control lymphoblast cell lines (Fig. 5A,B, top panels). We tested a range of ISRIB and PERKi concentrations and determined that low levels of these compounds could rescue the hypersuppression of translation observed in VWMD patient cell lines to the levels observed in healthy control cell lines during stress (Fig. 5). Cells from healthy controls recovered more translation activity in the presence of ISRIB or PERKi than cells from VWMD patients, supporting the idea that eIF2B activity is somewhat compromised in cells with *EIF2B2* mutations. These results indicate that eIF2B activity may be increased to normal levels seen in a stress response in VWMD cells by therapeutic intervention that either limits the levels of phosphorylated eIF2α or interferes with the interaction between phospho-eIF2α and eIF2B, such that VWMD stress-induced hypersuppression of translation is ameliorated.

### Cells with VWMD-causing *EIF2B2* (E213G/E304X) mutations are hypersensitive to chronic ER stress

**FIGURE 6.**
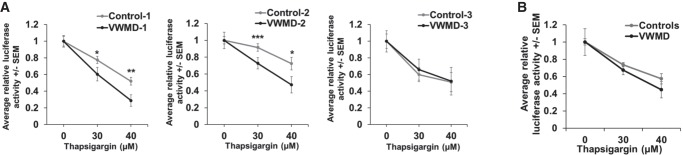
VWMD patient lymphocytes with *EIF2B2*^*E213G*^^/*E304X*^ mutations are hypersensitive to severe chronic thapsigargin stress. Immortalized lymphoblasts from matched control and VWMD patient cell lines with *EIF2B2* mutations were treated with 0, 30, or 40 µM thapsigargin for 24 h. Cellular ATP content was then measured as a proxy for cell viability. The results of each matched VWMD and Control cell line are presented in *A*, and the average viability of pooled controls and VWMD patient cell lines shown in *B*. The average ±SEM from four to six experiments is shown, and Student's *t*-test was used to assess significance, with (*) *P* ≤ 0.05, (**) *P* ≤ 0.01, and (***) *P* ≤ 0.005.

The defect in the ISR in the VWMD cell lines raised the possibility that cells with VWMD mutations might be more sensitive to stress conditions. To examine this possibility, we tested the ability of immortalized lymphocytes from VWMD patients or matched controls to survive in the presence of thapsigargin, as long-term ER stress causes cell death (Sano and Reed 2013). We observed that the VWMD patient cell lines harboring *EIF2B2* (E231G/E304X) mutations (VWMD-1 and VWMD-2) were hypersensitive to prolonged treatment with high amounts of thapsigargin (Fig. 6), as assessed by changes in cellular ATP content, an excellent proxy for cell viability (Petty et al. 1995). Interestingly, the “VWMD-3” cell line (*EIF2B2* [S171F/M203X]) was not more sensitive to chronic ER stress and was isolated from a patient with less severe disease progression than the hypersensitive cell lines from “VWMD-1” and “VWMD-2” patients (Fogli et al. 2003, 2004). We also observed that lower levels of thapsigargin did not cause an appreciable change in cell viability over 24 h in any of the cells lines (not shown). This suggests that cells with strong VWMD mutations are more sensitive to high levels of chronic stress, which might contribute to disease progression in patients (see discussion).

## DISCUSSION

A key issue in understanding VWMD is determining how the mutations in eIF2B subunits affect translation and thereby contribute to disease progression. Several observations from the literature and this work demonstrate that the mutations in *EIF2B* genes do not affect bulk translation in the absence of stress. Specifically, we observe that cells with VWMD mutations translate at similar levels as normal control cells (Fig. 1). Similar results have been seen with other VWMD alleles, and in different cellular contexts (Kantor et al. 2005; van Kollenburg et al. 2006b; Sekine et al. 2016; Wong et al. 2018). The normal levels of bulk translation in VWMD cells occur despite clear data that many VWMD mutations reduce the exchange rate of GDP for GTP on eIF2α (van Kollenburg et al. 2006b; Horzinski et al. 2010; Liu et al. 2011; Wortham and Proud 2015; Wong et al. 2018). This implies that under normal growth conditions the GDP–GTP exchange rate for eIF2α is not rate-limiting for bulk translation. Thus, the impact of VWMD on translation is not a general reduction in translation rate under all conditions, which is consistent with the birth and initial viability of classical VWMD patients.

We present several observations demonstrating that at least the VWMD mutations in *EIF2B2* examined herein alter the cellular response to stress by hyperrepression of translation during a strong stress, and a defect in the recovery of translation during the recovery phase of a stress response. First, we observed that VWMD cells hyperrepress translation in response to thapsigargin, DTT, or arsenite (Figs. 2, 3; Supplemental Figs. S1, S2). Second, we observed that during later time points in a stress response, control cells began to recover translation while VWMD cells did not (Fig. 2). Third, during recovery from thapsigargin or DTT stress we observed that VWMD cells recovered at slower rates and to a lower degree than control cells (Fig. 3; Supplemental Fig. S2). Fourth, we observed that the induction of the GADD34 phosphatase, which dephosphorylates eIF2α to contribute to translation restoration during stress recovery, is delayed and occurs to a lower extent during acute ER stress in VWMD patient cell lines (Fig. 4A,B), which explains the persistence of phospho-eIF2α during stress recovery in the VWMD cell lines (Fig. 4C,D). We interpret these observations to suggest that the VWMD variant eIF2B complex is sufficient in unstressed cells to enable adequate GTP exchange on eIF2α for bulk translation initiation. However, during stress when eIF2α is phosphorylated, the VWMD variant eIF2B forms are unable to allow sufficient GTP exchange on eIF2α, leading to hyperrepression of translation and perturbation of a negative feedback loop that relies on sufficient induction of *GADD34* gene expression during acute stress. This insight provides a possible mechanistic basis for classical VWMD pathogenesis wherein patients exhibit episodic white matter loss following trauma or illnesses that activate cellular stress response pathways in the brain.

Additional observations in the literature argue that other VWMD mutations lead to hypersuppression of translation during acute stress, although this affect has not been explicitly noted. Three previous studies demonstrated a stronger suppression of translation activity in *EIF2B* mutant cell lines during weak acute ER stresses that caused a reduction in global translation activity in control cells to only ∼30%–50% that of unstressed cells. First, fibroblasts derived from VWMD patients with *EIF2B4* and *EIF2B5* mutations on average displayed an almost significant (*P* = 0.0500005, Student's *t*-test) ∼50% reduction in translation activity compared to ∼40% suppression of translation in control cells (Kantor et al. 2005). Second, Sekine et al. (2016) assessed translation activity in *Eif2b4*-mutant CHO cells during an acute thapsigargin stress, and these cells also on average exhibited lower translation activity (∼60%–70% suppression in the mutant cell line versus ∼50% suppression in the wild-type cells). Finally, while DTT stress reduced translation to ∼30% in control 293T cell lines, translation was hyperrepressed to ∼15% in isogenic 293T cell lines carrying VWMD mutations in *EIF2B1*, *EIF2B4*, or *EIF2B5* (Wong et al. 2018). These results suggest that hyperrepression of translation during acute stress may be a common response in cells with VWMD mutations. This suggests a parsimonious model for how VWMD mutations propagate their effects wherein weak hypomorphic alleles of eIF2B are sufficient to allow normal translation, under conditions where eIF2B function is not limiting, but in specific cellular contexts, or stress responses, the diminished eIF2B function is not sufficient to allow residual translation during stress, leading to a failure to properly execute aspects of the ISR.

It has been noted that during a 30-min severe heat stress (44°C), translation in four different VWMD patient lymphoblasts was reduced to similar levels as a single healthy control cell pool (∼20% the activity in unstressed cells) (van Kollenburg et al. 2006b). However, it is possible that no differences were observed in this study because severe heat stress causes translational suppression through the inhibition of translation elongation (Shalgi et al. 2013), not by targeting of translation initiation mediated by eIF2B activity. Therefore, sensitivity to stresses that cause robust phosphorylation of eIF2α and the ISR could be a common mechanism of many VWMD cases that should be explored further in the future.

The ISR involves additional feedback loops that allow cells to adapt, particularly in chronic stress conditions. For example, during prolonged ER stress translation can resume even in the presence of continued eIF2α phosphorylation in a manner strongly dependent on eIF3 (Guan et al. 2017). Similarly, constitutive high expression of a misfolded protein in the ER leads to a compensatory ER expansion and an increase in the ER localized chaperone, BiP (Bakunts et al. 2017). Since these additional feedback loops will also require new protein synthesis during conditions of stress and eIF2 phosphorylation, VWMD mutations may also prevent these feedback loops from occurring due to reduced translation during the initial stress conditions, which can be examined in future experiments.

The observation that the two arms of the acute ISR response—translation repression and transcriptional activation of stress response genes—can be partially uncoupled by naturally occurring mutations in *EIF2B* genes that cause VWMD is important for two main reasons. First, white matter loss is a facet of many other neurodegenerative disorders including Alzheimer's disease, and occurs to some degree with normal aging (Double et al. 1996; Cardenas et al. 2007). Aberrant regulation of the ISR could be a generalizable mechanism for white matter loss in other myelin disorders. For example, mutations in the gene encoding CReP, a constitutively expressed phosphatase that dephosphorylates phospho-eIF2α in unstressed conditions and during acute ER stress (Jousse et al. 2003), cause developmental delays in white matter myelination in addition to microcephaly, short stature and learning disability (Kernohan et al. 2015). Dysregulation of the UPR and ER stress pathways may also contribute to several other genetic myelin disorders including Charcot–Marie–Tooth neuropathies (Volpi et al. 2017), indicating that targeting the ISR could serve as a useful treatment strategy for many diseases. It is also possible that dysregulation of the ISR might contribute to other aspects of VWMD including cataracts, as elevated levels of phospho-eIF2α are observed in human lens samples from patients with congenital cataracts (Yang et al. 2015). We suggest that ISRIB, PERK inhibitors, and potentially other small molecules that target the translational repression arm of the ISR could be used as a therapeutic option for VWMD, as we have demonstrated their utility in scaling back the translation repression threshold observed in patient cell lines during stress (Fig. 5). Moreover, recent work has provided additional biochemical evidence that ISRIB can rescue the function of eIF2B complexes with VWMD alleles (Wong et al. 2018). Since we observed that 1.25 nM ISRIB was sufficient in VWMD cell lines to restore translation during stress to control levels, one possible therapeutic approach may be to use very low doses of an ISRIB-like molecule to restore eIF2B function sufficiently during stress conditions, which might limit potential toxic side effects.

In addition to VWMD mutations causing a more robust and prolonged ISR, evidence in the literature suggests VWMD mutations will potentially affect translation in three other manners. First, one anticipates a lower level of GTP exchange activity caused by *EIF2B* mutations would suggest that cells with low eIF2α phosphorylation, which would not normally be sufficient to trigger a stress response in cells, might trigger the ISR. Evidence for this possibility comes from the observation that low levels of thapsigargin trigger a more robust induction of ATF4 in VWMD patient cells than control cells (Kantor et al. 2005). Second, the reduced guanine exchange factor activity due to VWMD might be low enough to fail to support full translation under conditions with increased demand for translation. In support of this possibility, in response to LPS treatment astrocytes in mouse models of VWMD fail to fully induce cytokines and chemokines needed for astrogliosis and remyelination (Cabilly et al. 2012). Finally, one has to anticipate that the translation rate of some specific mRNAs will be sensitive to even partial reduction in eIF2B function, and therefore VWMD cells will have alterations in their proteome, which is supported by an altered proteome in VWMD cells, and brains from VWMD mice as compared to control samples (Gat-Viks et al. 2015; Raini et al. 2017). This diversity of differences in translation suggests that VWMD could arise by a complex mixture of these different alterations. For example, the hyperrepression of translation during stress may trigger more cellular apoptosis and demyelination during trauma or infections, followed by an inability of astrocytes to properly activate astrogliosis and remyelination. Future examination of how these perturbations occur in animal models and/or in neuronal cells, particularly glia, derived from human inducible pluripotent stem cells and contribute to disease progression will be important, particularly considering that neuronal cell types could display a differential sensitivity to chronic stresses as compared to immortalized cell lines used in this and other studies.

Our results suggest that hypersuppression of translation disrupts a negative feedback loop wherein GADD34 is rapidly induced and dephosphorylates phospho-eIF2α to allow translation resumption for recovery or to engage in a chronic ER stress response in part through genes induced transcriptionally by CHOP, ATF3, and ATF4. Future work should aim to determine how VWMD patient cell lines respond to chronic stress and how the temporal regulation of stress-induced gene expression is affected by various *EIF2B* mutations. For example, a recent study (Sekine et al. 2016) demonstrated that mutations in *Eif2b4* analogous to those found in VWMD patients cause increased expression of a CHOP reporter protein after 24 h of chronic ER stress, and elevated levels of CHOP, BiP, and GADD34 mRNA were present in brain tissue from VWMD patients harboring mutations in *EIF2B5* and *EIF2B2* subunits (van Kollenburg et al. 2006a). However, other studies have concluded there are few differences in the levels of stress-induced genes in VWMD patient cells compared to controls (Horzinski et al. 2010). It should be determined if contradictory results in the literature regarding this problem are explained by effects of different VWMD-causing mutations, the time and severity of the stressor, or the cell type. A detailed analysis of the kinetics of stress-induced gene expression at the levels of transcription and translation over the course of acute and chronic ER stress responses could illuminate how changes in translation regulation during the acute phase of the ISR might impact the chronic stress response and/or identify any compensatory mechanisms that might facilitate a proper chronic stress response in the face of an aberrant acute stress response. Further, elucidating which stress-induced genes are affected at the level of transcription by global translation hypersuppression as occurs in these VWMD patient cell lines could yield important insights into how stress-induced genes are regulated for proper dynamic control of the stress response.

## MATERIALS AND METHODS

### Lymphoblast cultures

Immortalized lymphocytes from three VWMD patients and three age-, ethnicity- and sex-matched apparently healthy controls were obtained from the Coriell Biorepository (Table 1). Cells were maintained in RPMI 1640 (GibcoTM) with 15% fetal bovine serum (FBS; Atlas Biologicals) and 1% streptomycin/penicillin in suspension flasks at 37°C under 5% CO_2_.

**TABLE 1.**
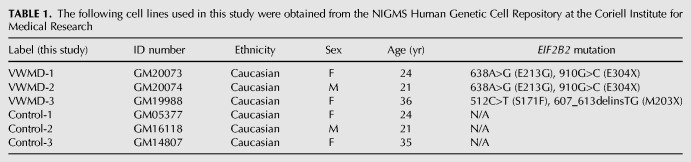
The following cell lines used in this study were obtained from the NIGMS Human Genetic Cell Repository at the Coriell Institute for Medical Research

### Western blotting

To assess specific protein levels, equal numbers of patient lymphoblasts or matched controls were incubated in the presence or absence of 1 µM thapsigargin for 0.5, 1, or 2 h at 37°C and lysed using NP-40 cell lysis buffer (50 mM Tris-HCl pH 8.0, 150 mM NaCl, 0.5% NP-40 substitute, 5 mM EDTA) or RIPA buffer (50 mM Tris-HCl pH 8, 150 mM NaCl, 1% NP-40 substitute, 0.5% deoxycholate, 0.1% SDS plus 0.1U/µL Turbo DNase) and phosphatase/protease inhibitor cocktail (Cell Signaling Technology). Protein concentrations were determined using the Bradford protein assay (Bio-Rad), and equal amounts of protein were loaded on 4%–12% NuPAGE protein gels (Thermo Fisher Scientific). Proteins were transferred to nitrocellulose membranes and probed for GAPDH (Cell Signaling Technology 3683), EIF2B1 (Thermo Fisher Scientific PA5-28992), EIF2B2 (Proteintech 50-555-431), EIF2B3 (Santa Cruz Biotechnology sc-166768), EIF2B4 (Santa Cruz Biotechnology sc-28855), EIF2B5 (H-9; Santa Cruz Biotechnology sc-514056), GADD34 (Proteintech 10449-1-AP), EIF2α (Cell Signaling Technology 9722), or phospho-EIF2α (Abcam ab32157) with anti-rabbit (Cell Signaling Technology 7074) or anti-mouse (Sigma-Aldrich A4416) HRP-conjugated secondary antibodies. Proteins were detected using SuperSignal West Dura extended duration or Femto Maximum sensitivity substrate (Thermo Fisher Scientific) for chemiluminescent imaging. Membranes were stripped using OneMinute Advance WB Stripping Buffer (GM Biosciences) to reprobe for loading controls. Relative protein abundances are reported from three to four independent experiments compared to GAPDH and relative phospho-eIF2α levels were determined relative to total eIF2α on the same blot.

### Metabolic labeling of nascent proteins during and after stress

Protein biosynthesis was assessed in human lymphoblasts in the presence and absence of stress using ^35^S-incorporated methionine and cysteine (EXPRE35S35S Protein Labeling Mix, PerkinElmer). Equal numbers (1 × 10^6^) of lymphoblasts were washed once with RPMI 1640 lacking cysteine and methionine (Sigma-Aldrich) with 15% dialyzed FBS (Sigma-Aldrich) and 1% streptomycin/penicillin and incubated for 15 min at 37°C to deplete internal amino acid stores. To assess translation activity during thapsigargin stress, enough thapsigargin (Calbiochem) to reach 0, 0.125, 0.25, 0.5, or 1 µM was added to each sample and incubated for 30 min at 37°C. Approximately 10 µCi ^35^S-labeled met/cys was added to each sample and incubated 30 min at 37°C prior to sample collection. For stress time-course experiments, cells were incubated in the presence or absence of 1 µM thapsigargin or 0.5 mM sodium arsenite, and metabolic labeling was done for 15 (thapsigargin stress) or 30 (arsenite stress) minutes immediately prior to sample collection. Translation recovery assays were done by incubating samples for 1 h in the presence or absence of 1 µM thapsigargin or 2 mM DTT in complete cys- and met-depleted RPMI then washing twice with complete medium via successive 5-min centrifugations at 500 rcf. Samples were then resuspended in complete cys-/met- depleted RPMI. Approximately 10 µCi ^35^S-labeled cys/met was added immediately after washing, or after 30 min to enable 30-min labeling periods for recovery after 30 min (“R-30”) and 60 min (“R-60”) post-stress. Cells were pelleted and stored at −80°C, then lysed in NP-40 lysis buffer with protease inhibitor cocktail (Sigma-Aldrich). Insoluble materials were removed by centrifugation and equal volumes of lysate were heat-denatured and run on 4%–12% NuPAGE protein gels (Thermo Fisher Scientific). Gels were exposed to phosphor screens and imaged using the Typhoon FLA 9500 phosphorimager. ImageJ was used to quantify signal intensity in each lane and the translation activity in each cell line in the presence of thapsigargin relative to the unstressed condition was determined. Results shown are the average ±SD of three or four independent experiments with significance assessed by Student's *t*-test.

### Drug treatments

To determine the effect of drugs acting on the PERK-eIF2 pathway on translation activity, equal numbers of lymphoblasts were either untreated, treated with 1 µM thapsigargin, or 1 µM thapsigargin plus *trans*-ISRIB (Fisher Scientific), or PERK inhibitor I (GSK2606414; EMD Millipore). Samples were incubated in the presence or absence of 1 µM thapsigargin and each drug as above for 1 h at 37°C and labeled with ^35^S- met and cys for 30 min immediately prior to collection. ISRIB was tested at 1.25, 2.5, 5, 10, and 100 nM and PERK inhibitor I tested at 12.5, 25, 50,100, and 1000 nM concentrations. The average ±SD of nascent protein signal from SDS-PAGE gels and phosphorimaging as described above from three independent experiments is reported. Significance was determined using Student's *t*-test.

### Lymphocyte viability assays

Equal numbers of immortalized lymphocytes were seeded into 96-well flat-bottom white plates in duplicate or triplicate wells in the presence or absence of thapsigargin (Calbiochem) at 30 or 40 µM. Cells were incubated for 24 h and ATP was measured as a proxy for cell viability using the Promega CellTiter-Glo 2.0 assay with the GloMax-Multi Detection System (Promega). The results of four or six independent biological replicates are reported ±SEM and significance was assessed by *t*-test.

## SUPPLEMENTAL MATERIAL

Supplemental material is available for this article.

## Supplementary Material

Supplemental Material
