# Supplementary material for: EIF2B2 mutations in vanishing white matter disease hypersuppress translation and delay recovery during the integrated stress response
Source: RNA. 2018 Jun;24(6):841–52. doi: 10.1261/rna.066563.118 (PMC5959252; doi:10.1261/rna.066563.118)
Supplement: Supplemental Material [file supp_24_6_841__index.html]

EIF2B2 mutations in vanishing white matter disease hypersuppress translation and delay recovery during the integrated stress response — Supplemental Material 

# *EIF2B2* mutations in vanishing white matter disease hypersuppress translation and delay recovery during the integrated stress response

## Supplemental Material

- Supplemental\_Fig\_S1.pdf
- Supplemental\_Fig\_S2.pdf
