# Supplementary material for: EIF2B2 mutations in vanishing white matter disease hypersuppress translation and delay recovery during the integrated stress response
Source: RNA. 2018 Jun;24(6):841–52. doi: 10.1261/rna.066563.118 (PMC5959252; doi:10.1261/rna.066563.118)
Supplement: Supplemental Material [file supp_066563.118_Supplemental_Fig_S1.pdf]

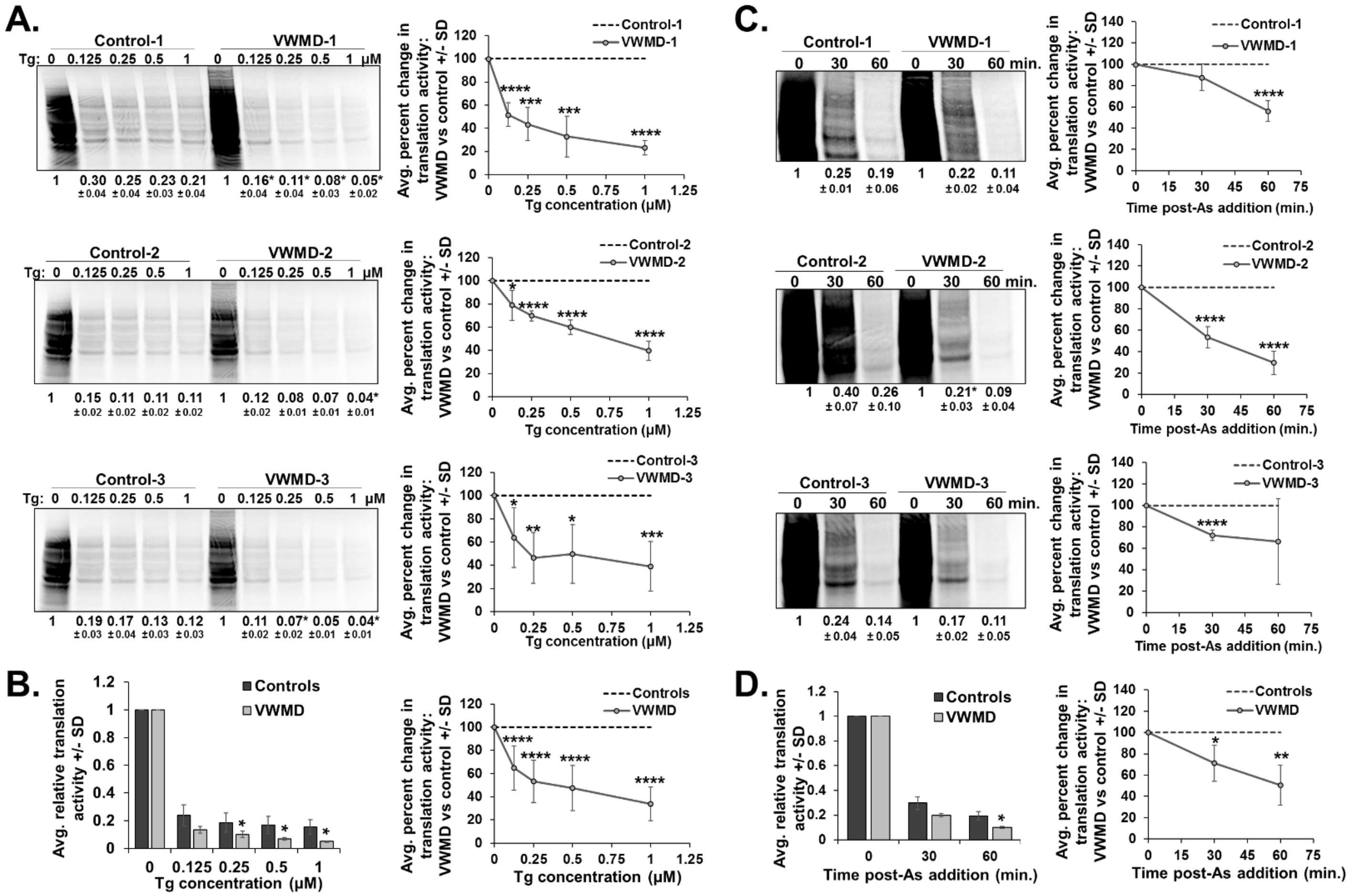

Figure S1. Cell lines from VWMD patients have reduced translation activity under stress caused by varying concentrations of thapsigargin or over an arsenite stress timecourse. A) Equal numbers of lymphoblasts from VWMD patients or matched controls were exposed to 0, 0.125, 0.25, 0.5 or 1 uM thapsigargin for 1 hour and pulse-labeled with 35S-met and -cys for 30 minutes prior to collection. Equal volumes of cell lysates were run on SDS-PAGE gels and phosphor-imaging performed to detect nascent proteins. Representative phosphor- images are shown at left and the average relative lane intensity +/- SEM from three experiments is depicted below them. The average percent change in translation activity +/- SD in VWMD patient cell lines compared to controls is shown graphically in the panels at right. B) The average relative translation activity +/- SD (left) and the average percent change in translation activity in all pooled VWMD patient cells relative to all pooled control cell lines (right) treated with thapsigargin at the indicated concentrations. C) Cells from VWMD patients or healthy controls were treated with 0.5 mM arsenite for 30 or 60 minutes and pulse labeled as in (A) for 30 minutes with 35S-met and -cys prior to collection. D) The average relative translation activity +/- SD (left) and average percent difference in translation activity +/- SD in all pooled VWMD patient cell lines versus pooled control cell lines (right) during 30 or 60 minutes or arsenite stress. Results represent three independent experiments. Student's t-test was done to assess significance between translation activity in VWMD and control patient lines during stress with * indicating $p < 0.05$; ** $p < 0.01$; *** $p < 0.005$ and **** $p < 0.001$.
