# Supplementary material for: EIF2B2 mutations in vanishing white matter disease hypersuppress translation and delay recovery during the integrated stress response
Source: RNA. 2018 Jun;24(6):841–52. doi: 10.1261/rna.066563.118 (PMC5959252; doi:10.1261/rna.066563.118)
Supplement: Supplemental Material [file supp_066563.118_Supplemental_Fig_S2.pdf]

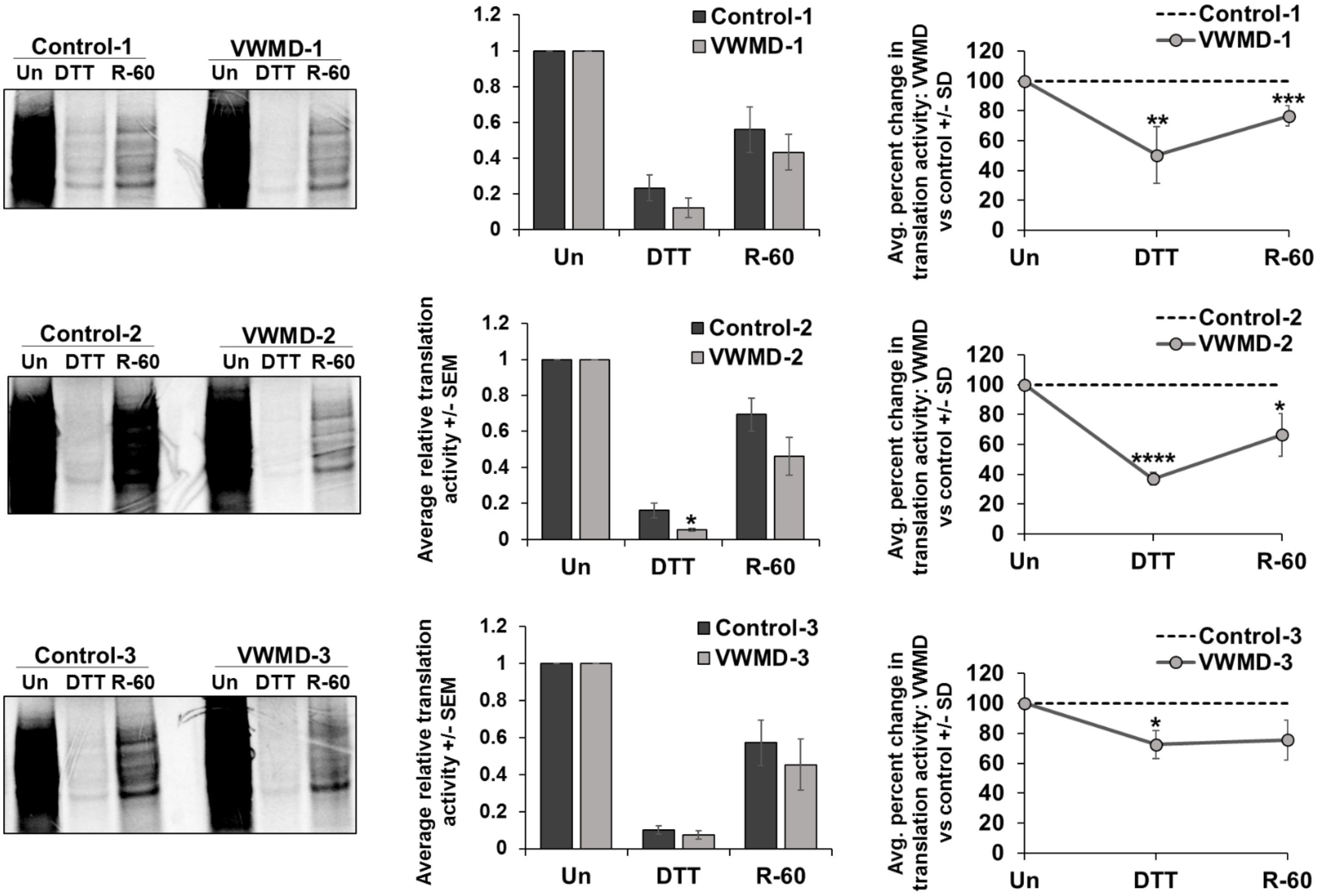

Figure S2. Lymphoblasts from VWMD patients exhibit hyper-suppression of translation during and after acute DTT stress. Equal numbers of lymphoblasts from VWMD or healthy controls were untreated “Un” or exposed to 2 mM DTT for 60 minutes “DTT” and collected or washed twice and allowed to recover for 60 minutes “R-60”. Cells were pulse-labeled with 35S-met and -cys for 30 minutes prior to collection, lysed and equal volumes lysate run on SDS-PAGE gels and exposed to phosphor screens. Representative images are shown at left with the average relative translation activity +/- SEM shown in the bar graphs and the average percent difference in translation activity in the VWMD patient cell lines compared to controls shown in the line graphs at right. Results represent 3-4 independent experiments and Student’s t-test was used to assess significance with * indicating $p < 0.05$; ** $p < 0.01$; *** $p < 0.005$ and **** $p < 0.001$.
